# Assessment of Conjunctival Microvascular Hemodynamics in Stages of Diabetic Microvasculopathy

**DOI:** 10.1038/srep45916

**Published:** 2017-04-07

**Authors:** Maziyar M. Khansari, Justin Wanek, Michael Tan, Charlotte E. Joslin, Jacob K. Kresovich, Nicole Camardo, Norman P. Blair, Mahnaz Shahidi

**Affiliations:** 1 Department of Ophthalmology and Visual Sciences, University of Illinois at Chicago, Chicago, IL, 60612, USA; 2 Department of Bioengineering, University of Illinois at Chicago, Chicago, IL, 60607, USA; 3 Division of Epidemiology and Biostatistics, School of Public Health, University of Illinois at Chicago, Chicago, IL 60612, USA; 4 Population Health, Behavior and Outcomes Program, University of Illinois Cancer Center, Chicago, IL 60612, USA; 5 Department of Ophthalmology, University of Southern California, Los Angeles, CA 90033, USA

## Abstract

Diabetes impairs the microcirculation and function of various vital tissues throughout the body. The conjunctival microcirculation can be non-invasively imaged and thus enables assessment of microvascular hemodynamics. In this study, alterations in conjunctival microvascular hemodynamics were quantitatively assessed at stages of increasing diabetic microvasculopathy based on diabetic retinopathy (DR). Subjects were categorized into non-diabetic control (C, N = 34), no clinically visible DR (NDR, N = 47), non-proliferative DR (NPDR, N = 45), and proliferative DR (PDR, N = 35). Conjunctival hemodynamic descriptors, namely vessel diameter (D), blood velocity (V), blood flow (Q), wall shear rate (WSR), and wall shear stress (WSS) were measured in arterioles and venules, and compared between DR and C subjects using generalized linear mixed models. In arterioles, V, WSR, and WSS were lower in NDR (P ≤ 0.01). V was lower in NDR than NPDR and PDR subjects (P ≤ 0.02). In venules, D was higher in NDR and NPDR (P ≤ 0.03), while V was lower in PDR (P = 0.04). Venular V and Q were higher in NPDR than PDR subjects (P ≤ 0.04). WSR and WSS were lower in all stages of DR (P ≤ 0.05), suggestive of the potential of WSS as a marker of diabetic microvasculopathy. Quantitative assessment of conjunctival hemodynamics can potentially be useful for evaluation of diabetic microvasculopathy.

Diabetes was the seventh leading cause of death in the US in 2010^1^. The prevalence of diabetes among US adults is projected to increase from 14% in 2010 to 21% by 2050^2^, representing a significant burden on the population. Previous studies have reported a high prevalence of microvascular disease in diabetic subjects^3^,^4^, and indeed the most common cause of morbidity and mortality among diabetics is related to vasculopathy^5^,^6^. Alterations in circulation due to diabetes adversely affect various organ systems, causing complications such as, diabetic retinopathy (DR), nephropathy, neuropathy, cardiovascular disease, genitourinary problems, amputations and foot ulcers^7^. Therefore, assessment of microvascular hemodynamics can be useful for evaluation and monitoring of complications due to diabetes.

Microvascular hemodynamic alterations due to diabetes have been reported in various tissues, including the brain, heart, foot, sublingual tissues, nail fold, retina, and conjunctiva^8^,^9^,^10^,^11^,^12^,^13^,^14^,^15^,^16^,^17^,^18^,^19^,^20^,^21^,^22^,^23^,^24^,^25^,^26^. Due to the accessibility of the conjunctiva for direct visualization of microcirculation, alterations in the conjunctival microvasculature due to diabetes have been reported based on determination of a severity index (SI)^21^,^23^,^24^,^25^. The SI incorporated several factors, including the number of blood vessels with abnormal morphometry, blood vessel diameter, arteriole to venule ratio, blood velocity and viscosity. However, these studies did not provide assessment of conjunctival hemodynamic alterations at progressive stages of diabetic microvasculopathy.

Since DR stage is thought to parallel progressive levels of diabetic microvasculopathy in other tissues^27^,^28^, assessment of the conjunctival hemodynamics at stages of DR may become useful for gaining a better understanding of diabetes pathophysiology, and potentially allow diagnostic evaluation of diabetic microvasculopathy. Additionally, conjunctival and retinal hemodynamics may be comparable as suggested by a previous report of similarities in diabetic-related microvasculopathies between the conjunctiva and the retina^20^. The purpose of the current study was to provide a comprehensive and quantitative assessment of alterations in conjunctival hemodynamic descriptors at progressive stages of diabetic microvasculopathy by application of our previously established conjunctival microcirculation imaging technique^29^.

## Methods

### Subjects

The study was approved by an institutional review board of the University of Illinois at Chicago. The study was explained to subjects and written informed consents were obtained from participants according to the tenets of the Declaration of Helsinki. The cohort consisted of 161 subjects (58 males and 103 females) with ages ranging from 21 to 87 years. Based on a complete clinical history and ocular examination, including a dilated fundus examination, the subjects were categorized into one of four groups: non-diabetic control (C, N = 34) and 3 diabetic groups of increasing microvasculopathy severity: no diabetic microvasculopathy corresponding to no clinically visible diabetic retinopathy (NDR, N = 47), mild/moderate diabetic microvasculopathy corresponding to non-proliferative diabetic retinopathy (NPDR, N = 45), and advanced diabetic microvasculopathy corresponding to proliferative diabetic retinopathy (PDR, N = 35). Twelve (5 NDR, 2 NPDR, 5 PDR) and 115 (42 NDR, 43 NPDR, 30 PDR) subjects had type 1 and 2 diabetes, respectively. Exclusion criteria were inability to give informed consent or participate in the study, stroke or myocardial infarction within 3 months of imaging, active angina, dry eye syndrome, conditions that can affect the ocular surface, clinical diagnosis of glaucoma, age-related macular degeneration, retinal vascular occlusions or any other retinal, choroidal or optic nerve disease that could interfere with the staging of DR, history of intraocular surgery within 4 months of imaging, or cataract surgery within 4 months of imaging. Glycated hemoglobin (HbA1c), hematocrit (HCT), systolic blood pressure (SBP), diastolic blood pressure (DBP), and heart rate (HR) were measured at the time of imaging. Mean arterial pressure (MAP) was computed as (SBP + 2DBP)/3. Data from one eye per subject was included in the study based on the exclusion criteria, ability to maintain fixation during imaging, and image quality. If both eyes qualified, the eye with the larger number of acquired images was selected. During imaging, a headrest and forehead support were used to stabilize subject’s head, and a fixation target was presented to the fellow eye to minimize eye motion. Subjects were asked to suspend blinking during the one-second duration of image acquisition, and then allowed to blink normally.

### Image Acquisition

Imaging of the conjunctival microcirculation was performed using our previously described non-invasive optical imaging system (EyeFlow)^29^. Briefly, the imaging system comprised a slit lamp biomicroscope and a digital charged coupled device (CCD) camera (Prosilica GT, AVT, Exton, PA) for acquisition of image sequences of red blood cell motion through the conjunctival microvasculature. The slit lamp light source, fitted with a narrow band optical filter with a transmission wavelength of 540 ± 5 nm, was used to illuminate the conjunctival microvasculature. One-second image sequences were captured from the superficial conjunctival microvasculature at a rate of 50 frames per second with 5.1X magnification. Each image consisted of 1360 × 550 pixels with a pixel resolution of 1.25 μm on the object plane. This process was repeated to acquire image sequences from multiple non-overlapping conjunctival microvascular regions temporal to the limbus that encompassed up to 10 mm × 13 mm areas.

### Image Processing and Analysis

Conjunctival image sequences were automatically analyzed with our previously validated method^29^ using customized software written in MATLAB (Release 2015b, MathWorks, Inc. Natick, MA, USA). The automated method for measuring conjunctival hemodynamics consisted of several steps including image registration, vessel segmentation, centerline and bifurcation extraction, diameter measurement, blood flow detection, and axial blood velocity measurement. Briefly, an intensity-based image registration algorithm was employed to correct for eye movement in image sequences. Frames with blinks, large eye motion, or illumination artifacts were eliminated from each image sequence, then the longest consecutive number of frames were registered. A time-averaged image was then generated from the registered image set, and Frangi filtering was performed for segmentation of conjunctival vessels. Vessel centerlines were extracted by thinning the segmented vessels, then bifurcation points were identified to define centerlines of all individual vessel segments. To ensure adequate sampling and reliability of measurements, only vessel segments with centerline lengths above 50 microns were included for hemodynamic analysis. Variance filtering was performed on the remaining vessel segments to distinguish vessels with detectable blood flow. Vessel diameter (D) and vessel boundaries were measured by computing the full width at half maximum of intensity profiles, established perpendicular to the centerline direction at every five pixels along the microvessel. Axial blood velocity (V) was determined by tracking the movement of red blood cells along the vessel centerline in the registered image sequences using a spatial-temporal image (STI) technique^29^. The STI showed variation of intensity values along a vessel centerline over time due to red blood cell motion. V was calculated by determining the slope of the prominent bands in the STI. Average cross sectional velocity (V_s_) from D and V, blood flow (Q = V_s_πD^2^/4), wall shear rate (WSR = 8V_s_/D), dynamic blood viscosity (η) from HCT and D, and wall shear stress (WSS = ηWSR) were determined using previously described formulas^30^,^31^.

Blood vessels were categorized as arterioles or venules by visualization of the direction of blood flow within the vessels and distinguished if the flow diverged into smaller vessel branches (arteriole) or collected into a larger vessel (venule). If multiple measurements were obtained along the same vessel, data from the vessel segment with the longest centerline were included for analysis. Image acquisition was not synchronized with the heart rate, and hence arteriolar and venular hemodynamic measurements were obtained at different time points during the cardiac cycle, though hemodynamics in venules are less dependent on the cardiac cycle^32^.

### Statistical Analysis

Compiled data consisting of one value per hemodynamic descriptor (D, V, Q, WSR, WSS) per vessel per subject were analyzed using Stata version 12 (College Station, TX: StataCorp LP). Demographic and systemic physiologic data were compared among groups using the Chi-Square test or ANOVA. Mean conjunctival hemodynamic descriptors were computed and compared among C and stages of DR (NDR, NPDR and PDR) using ANOVA. A generalized linear mixed model (GLMM) with random intercepts was used to estimate beta (β) and 95% confidence intervals (CI) and examine associations between DR stage and each hemodynamic descriptor outcome. Fixed effects were analogous to standard regression analysis and estimated directly. The model assumed a Gaussian error distribution. Unadjusted models regressed the DR stage group (categorical) on the hemodynamic descriptors with no additional fixed effects. The random intercepts were established by identification of the individual study participants using their study id number. The adjusted models regressed the DR stage group (categorical), and the following fixed effects: age (continuous), race (categorical), sex (categorical), MAP (continuous), HR (continuous), HCT (continuous) and HbA1C (continuous) on the hemodynamic descriptors. Again, the random intercepts were established by identification of the individual study participants using their study id number. Since race was not matched between groups of subjects, race differences were adjusted in the models according to well-established statistical data analysis methodologies. Eye examined was not considered as a covariate in the model because it was not associated with hemodynamic descriptors. The association between V (dependent variable) and D (independent variable) was determined in each group and compared to C subjects while accounting for multiple measurements per subject in both adjusted and unadjusted models. The estimated β value (denoted by slope) derived by the model represented the increase in V per one unit increase in D. Statistical tests were two-sided and significance was accepted at P ≤ 0.05.

## Results

### Demographic and Physiologic Data

Subjects’ demographics and physiologic data are reported in Table 1. Sex, MAP, and eyes examined were not different among DR stages (P ≥ 0.3). However, age, race, HR, HCT, and HbA1C were different (P ≤ 0.03).

### Conjunctival Hemodynamic Descriptors in Arterioles

Conjunctival hemodynamic measurements were obtained in a total of 1861 arterioles. The mean (±standard deviation (SD)) number of arteriole measurements was 13 ± 8, 12 ± 6, 12 ± 5, and 10 ± 7 in C, NDR, NPDR, and PDR subjects, respectively. There was no difference in the number of arteriole measurements among the groups of subjects (P = 0.3).

Mean and SD of unadjusted conjunctival arteriolar D, V, Q, WSR, and WSS stratified by DR stage are provided in Table 2. D and Q were similar (P ≥ 0.8), while V, WSR, and WSS were different among DR groups (P ≤ 0.03).

Estimates of DR stage differences from the statistical model with and without adjusting for age, race, sex, MAP, HR, HCT, and HbA1C are shown in Table 3. D and Q were not different between C and stages of DR with and without adjusting for covariates (P ≥ 0.3). V, WSR, and WSS were lower in NDR than C subjects with and without adjusting for covariates (P ≤ 0.01). Additionally, unadjusted WSR and WSS were lower in NPDR than C subjects (P ≤ 0.04), but the adjusted differences were not significant (P ≥ 0.2). Similarly, unadjusted WSS was lower in PDR than C subjects (P = 0.05), but not after adjusting for covariates (P = 0.3). After adjusting for covariates, V and WSR were lower in NDR as compared to NPDR subjects (P ≤ 0.02; results not shown in Table 3) and V was lower in NDR as compared to PDR subjects (P = 0.02; results not shown in Table 3).

The associations between conjunctival arteriolar V and D stratified by DR stage with and without adjusting for age, race, sex, MAP, HR, HCT, and HbA1C are provided in Table 4. After adjusting for covariates, the associations between V and D were significant in C, NDR, and PDR. The associations between V and D were weaker in NPDR and PDR as compared to C subjects (P ≤ 0.006).

### Conjunctival Hemodynamic Descriptors in Venules

Conjunctival hemodynamic measurements were obtained in a total of 9027 venules. The mean (±SD) number of venule measurements was 52 ± 21, 59 ± 18, 59 ± 23, and 52 ± 16 in C, NDR, NPDR, and PDR subjects, respectively. There was no difference in the number of venules measurements among the groups of subjects (P = 0.1).

Mean and SD of unadjusted conjunctival venular D, V, Q, WSR, and WSS stratified by DR stage are provided in Table 5. D, V and Q were similar among groups (P ≥ 0.08), whereas WSR and WSS were different (P ≤ 0.05).

Estimates of DR stage differences from the statistical model with and without adjusting for age, race, sex, MAP, HR, HCT, and HbA1C are shown in Table 6. Q was not different between C and stages of DR with and without adjusting for covariates (P ≥ 0.1). D was higher in NDR than C subjects with and without adjusting for covariates (P = 0.03). WSR and WSS were lower in NDR than C subjects with and without adjusting for covariates (P ≤ 0.01). D was higher in NPDR than C subjects, regardless of the effects of age, race, sex, MAP, HR, HCT, and HbA1C (P ≤ 0.02). WSR was lower in NPDR than C subjects after adjusting for covariates (P = 0.05). WSS was lower in NPDR than C subjects with and without adjusting for covariates (P ≤ 0.02). V was lower in PDR than C subjects after adjusting for covariates (P = 0.04). WSR and WSS were lower in PDR than C subjects with and without adjusting for covariates (P ≤ 0.03). After adjusting for covariates, V and Q were higher in NPDR as compared to PDR subjects (P ≤ 0.04; results not shown in Table 6).

The associations between conjunctival venular V and D stratified by DR stage with and without adjusting for age, race, sex, MAP, HR, HCT, and HbA1C are provided in Table 7. After adjusting for covariates, the associations between V and D were significant in all groups. The association between V and D was stronger in NPDR as compared to C subjects (P = 0.01).

## Discussion

In the current study, a comprehensive and quantitative assessment of alterations in hemodynamic descriptors (D, V, Q, WSR, and WSS) within the conjunctival microcirculation network was reported differentially in arterioles and venules at progressive stages of DR. In arterioles, V was reduced in NDR subjects, consistent with a previous finding^22^, though arterioles and venules were not differentiated in this study. Arteriolar WSR were reduced only in NDR subjects, suggestive of a potential early marker of diabetic microvasculopathy. Conjunctival microvascular hemodynamic abnormalities were more frequent in venules than arterioles, similar to a previous report that used a non-quantitative method^26^. In venules, vasodilation was observed in NDR and NPDR subjects, consistent with previously studies^22^,^33^, though these studies did not differentiate dilation in arterioles and venules and reported vasodilation in the entire conjunctival microvascular network. Increased vascular endothelial growth factor (VEGF) expression is known to cause vasodilation^34^,^35^ and VEGF expression has been previously reported to be elevated in conjunctival macrophages, epithelial, endothelial, and fibroblast cells in NPDR and PDR subjects^36^. Therefore, the finding of venular vasodilation in NPDR may be attributed, at least in part, to the elevation of VEGF expression. Further combined studies of vascular caliber and VEGF levels are needed to investigate the potential effect of VEGF expression on conjunctival vasodilation. In venules, WSR was reduced at all stages of DR, which is likely attributed to the observed vasodilation in NDR and NPDR subjects, and decreased V in PDR subjects. Reduction in V is supported by previously reported increased blood viscosity in diabetic subjects^37^.

There is no previous study, to the best of our knowledge, that reported alterations in conjunctival Q in a quantitative manner due to diabetes. In the current study, no alteration in conjunctival Q in arterioles or venules of DR subjects was detected as compared to C subjects. This finding is in agreement with a previous study that compared the nail fold microcirculation between diabetic and non-diabetic subjects^38^. However, previous studies of the retinal circulation have reported conflicting results of increased Q in early DR^8^, unaltered Q in NDR or early DR^9^, decreased Q in NPDR^10^, decreased Q in PDR^11^, and unaltered Q in PDR^12^. Future studies are needed to evaluate Q in both retina and conjunctiva of the same subjects to determine whether conjunctival and retinal Q are related.

WSS is an important hemodynamic parameter in cardiovascular pathophysiology^39^ and affects endothelial functions, such as migration of leukocytes, adhesion, control of vessel diameter, cytoskeletal structure, and energy metabolism^39^,^40^,^41^,^42^. WSS is generally lower in subjects at risk of vascular diseases^43^, and causes vessel wall remodeling and pathophysiology^44^,^45^. Reduced WSS in the retinal arterioles of subjects with early DR^46^ and in the carotid and branchial arteries of diabetic subjects^47^,^48^ was previously reported. No previous study, to our knowledge, has reported WSS in conjunctival microcirculation of diabetic subjects. In the current study, WSS was lower in conjunctival arterioles of NDR subjects and in venules at all stages of DR, as compared to non-diabetic subjects.

Reduced WSS may promote endothelial dysfunction^46^ and contribute to the development of microvasculopathy and DR^49^,^50^. Furthermore, previous studies have found an association of reduced WSS with increased vascular cellular adhesion molecules-1 (VCAM-1)^51^,^52^ and upregulation in the expression of VCAM-1 and intercellular adhesion molecules-1 (ICAM-1) in DR which leads to leukocytes accumulation in the retinal microcirculation^53^,^54^,^55^. Therefore, assessment of WSS in the conjunctival microcirculation may be potentially useful for evaluating microcirculatory abnormalities due to diabetes with and without clinical DR.

Murray’s law^56^ predicts a linear relationship between V and D under a normal physiological condition. The large sample size in the current study allowed us to test the linearity of this relationship in the conjunctival microcirculation. A positive linear association was found between V and D in both arterioles and venules in C subjects. This finding is in agreement with a previous study that showed a trend of increased V with larger D^57^. Furthermore, alterations in the dependence of V on D were present in NPDR and PDR subjects that suggest physiological abnormalities in conjunctival arterioles and venules at clinical stages of DR.

In the current study, the number of venules was greater than arterioles which is primarily due to the conjunctiva anatomy in which arterioles are less numerous than venules, as previously reported^23^,^24^. The lower sampling of arterioles could also be attributed by the fact that arterioles tend to have lower image contrast compared to venules. Despite the difference in vessel sampling, the findings of the current study are based on a very large sample size of approximately ten thousand arterioles and venules.

There were limitations in the current study. First, the imaging system was not synchronized with the cardiac cycle to account for velocity changes in arterioles due to pulsatility which was reported previously^32^. However, variability of V measurements due to pulsatility was reduced by averaging multiple arteriole measurements per subject. In the future, synchronization of imaging system with the cardiac cycle will enable assessment of conjunctival hemodynamics at peak systolic and diastolic blood pressure and should improve reliability of arteriole measurements. Second, identification of arterioles and venules was performed manually. Although human error in the identification of vessel type cannot be completely eliminated, the error was likely minimal since the direction of blood flow was clearly visualized in the image sequences. Third, motion of red blood cells was detectable in superficial vessels that were in the focal plane of the instrument and between 6 and 75 microns in diameter.

In summary, in non-clinical DR, arteriolar V was decreased and venular D was increased, while venular V was decreased in advanced DR and D was increased in clinical DR. At all stages of DR, venular WSS was decreased. Future studies are needed to determine the association between retinal and conjunctival hemodynamic alterations and substantiate the value of conjunctival microcirculation imaging as a surrogate for screening and monitoring of DR. Additionally, further investigation is warranted to relate alterations in conjunctival microvascular hemodynamic descriptors with incidence of complications due to diabetic microvasculopathy. Overall, assessment of conjunctival hemodynamic alterations has the potential for diagnostic evaluation and longitudinal monitoring of diabetic microvasculopathy complications.

## Additional Information

**How to cite this article**: Khansari, M. M. *et al*. Assessment of Conjunctival Microvascular Hemodynamics in Stages of Diabetic Microvasculopathy. *Sci. Rep.* **7**, 45916; doi: 10.1038/srep45916 (2017).

**Publisher's note:** Springer Nature remains neutral with regard to jurisdictional claims in published maps and institutional affiliations.

## Figures and Tables

**Table 1:** Subjects’ demographics and physiologic data.

	Total (N = 161)		C (N = 34)	NDR (N = 47)	NPDR (N = 45)	PDR (N = 35)	P-value
	N %	%	%	%	%	%	
Sex							0.3^a^
Male	58	36	24	34	40	46	
Female	103	64	76	66	60	54	
Race							<**0**.**001**^a^
AA	74	46	12	62	51	51	
White	48	30	77	21	16	14	
Hispanic	39	24	12	17	33	34	
Eye Examined							0.8^a^
Right	108	67	74	64	64	69	
Left	53	33	27	36	36	31	
Age (years)	57 ± 12		61 ± 11	55 ± 14	58 ± 10	53 ± 9	**0**.**03**^b^
MAP (mmHg)	91 ± 13		89 ± 10	92 ± 11	91 ± 13	94 ± 17	0.4^b^
Heart Rate (bpm)	75 ± 11		69 ± 9	73 ± 10	78 ± 12	78 ± 11	**0**.**001**^b^
Hematocrit (%)	41 ± 6		44 ± 5	42 ± 5	40 ± 5	37 ± 6	<**0**.**001**^b^
HbA1C (%)	7.4 ± 1.9		5.5 ± 0.5	7.4 ± 1.5	8.4 ± 1.7	8.2 ± 2	<**0**.**001**^**b**^
HbA1C (mmol/mol)	57 ± 21		37 ± 6	57 ± 16	68 ± 19	66 ± 22	<**0**.**001**^**b**^

AA, MAP and HbA1C are abbreviations for African American, mean arterial pressure and hemoglobin A1C, respectively. C, NDR, NPDR, and PDR are abbreviations for non-diabetic control, no clinically visible diabetic retinopathy, non-proliferative diabetic retinopathy, and proliferative diabetic retinopathy, respectively. Bold indicates statistical significance. The ± symbol refers to standard deviation (SD).

P-values derived by Chi square (^a^) or ANOVA (^b^).

**Table 2:** Conjunctival hemodynamic descriptors in arterioles stratified by DR stage.

Hemodynamic Descriptor	C (N = 34)	NDR (N = 47)	NPDR (N = 45)	PDR (N = 35)	P-value*
D (μm)					
Mean ± SD	18 ± 5	19 ± 4	18 ± 4	18 ± 5	0.9
[Min–Max]	[6–61]	[7–53]	[7–47]	[7–58]	
V (mm/s)					
Mean ± SD	0.70 ± 0.23	0.54 ± 0.22	0.62 ± 0.24	0.64 ± 0.27	**0.03**
[Min–Max]	[0.08–3.02]	[0.07–3.24]	[0.07–3.27]	[0.08–3.44]	
Q (pl/s)					
Mean ± SD	144 ± 118	124 ± 98	135 ± 90	146 ± 115	0.8
[Min–Max]	[5–1672]	[3–2306]	[4–1234]	[3–1566]	
WSR (s^−1^)					
Mean ± SD	280 ± 115	193 ± 87	232 ± 109	245 ± 106	**0.003**
[Min–Max]	[15–2288]	[18–1310]	[15–1365]	[24–1563]	
WSS (dyne/cm^2^)					
Mean ± SD	8.6 ± 5.0	5.4 ± 3.2	6.2 ± 3.3	6.6 ± 3.7	**0.003**
[Min–Max]	[0.03–3.2]	[0.03–6.3]	[0.03–5.0]	[0.04–6.0]	
Arterioles sample size					
Mean ± SD	13 ± 8	12 ± 6	12 ± 5	10 ± 7	0.3
[Min–Max]	[4–32]	[4–33]	[4–27]	[3–31]	

C, NDR, NPDR, and PDR are abbreviations for non-diabetic control, no clinically visible diabetic retinopathy, non-proliferative diabetic retinopathy, and proliferative diabetic retinopathy, respectively. D, V, Q, WSR, and WSS are abbreviations for diameter, axial blood velocity, blood flow, wall shear rate and wall shear stress, respectively. Bold indicates statistical significance. The ± symbol refers to standard deviation (SD).

*P-value determined by ANOVA.

**Table 3:** Comparison of conjunctival hemodynamic descriptors in arterioles between C and DR subjects in unadjusted (Model 1) and adjusted (Model 2) models.

	D (μm)		V (mm/s)		Q (pl/s)		WSR (s^−1^)		WSS (dyne/cm^2^)	
	β	P	β	P	β	P	β	P	β	P
Intercept	18	<0.001	0.69	<0.001	144	<0.001	278	<0.001	8.6	<0.001
**Model 1**: DR Stage Effect*										
C	Ref.	Ref.	Ref.	Ref.	Ref.	Ref.	Ref.	Ref.	Ref.	Ref.
NDR	0.7	0.5	−0.16	**<0.001**	−21	0.3	−87	**<0.001**	−3.2	**<0.001**
NPDR	0.7	0.4	−0.07	0.2	−6	0.8	−47	**0.04**	−2.4	**0.01**
PDR	0.4	0.7	−0.04	0.4	8	0.7	−32	0.2	−1.8	**0.05**
Intercept	21	<0.001	0.98	<0.001	575	<0.001	755	<0.001	19.3	<0.001
**Model 2**: DR Stage Effect**										
C	Ref.	Ref.	Ref.	Ref.	Ref.	Ref.	Ref.	Ref.	Ref.	Ref.
NDR	0.1	0.9	−0.11	**0.01**	−16	0.4	−60	**<0.001**	−2.2	**<0.001**
NPDR	0.8	0.4	0.02	0.7	14	0.5	−15	0.5	−1.1	0.2
PDR	0.9	0.4	0.00	0.1	13	0.6	−18	0.4	−0.9	0.3

C, NDR, NPDR, and PDR are abbreviations for non-diabetic control, no clinically visible diabetic retinopathy, non-proliferative diabetic retinopathy, and proliferative diabetic retinopathy, respectively. D, V, Q, WSR, and WSS are abbreviations for diameter, axial blood velocity, blood flow, wall shear rate and wall shear stress, respectively. Bold indicates statistical significance.

*Unadjusted.

**Adjusted for age, race, sex, mean arterial pressure, heart rate, hematocrit, and hemoglobin A1C.

**Table 4:** Comparison of association between conjunctival V and D in arterioles between C and DR subjects in unadjusted (Model 1) and adjusted (Model 2) models.

	Slope (s^−1^)	95% CI	P-value*
**Model 1**: **DR Stage Effect**			
C	3	−4–9	Ref
NDR	**11**^a^	**6**–**15**	**0**.**05**^b^
NPDR	3	−2–9	0.9
PDR	5	−1–11	0.5
**Model 2**: **DR Stage Effect**			P-value**
C	**15**^a^	**7**–**22**	Ref
NDR	**19**^a^	**14**–**24**	0.1
NPDR	5	−1–11	**0.001^b^**
PDR	**10**^a^	**3**–**17**	**0**.**006**^b^

Slopes of the regression lines relating V and D are provided. C, NDR, NPDR, and PDR are abbreviations for non-diabetic control, no clinically visible diabetic retinopathy, non-proliferative diabetic retinopathy, and proliferative diabetic retinopathy, respectively. Bold indicates statistical significance.

^a^Significant association between V and D.

^b^Association between V and D significantly different than C (ref).

*Unadjusted.

**Adjusted for age, race, sex, mean arterial pressure, heart rate, hematocrit, and hemoglobin A1C.

**Table 5:** Conjunctival hemodynamic descriptors in venules stratified by DR stage.

Hemodynamic Descriptor	C (N = 34)	NDR (N = 47)	NPDR (N = 45)	PDR (N = 35)	P-value*
D (μm)					
Mean ± SD	20 ± 2	21 ± 3	21 ± 3	20 ± 3	0.08
[Min–Max]	[6–75]	[7–71]	[7–68]	[7–74]	
V (mm/s)					
Mean ± SD	0.59 ± 0.17	0.54 ± 0.17	0.57 ± 0.13	0.52 ± 0.19	0.3
[Min–Max]	[0.06–4.55]	[0.07–4.39]	[0.05–6.34]	[0.04–4.9]	
Q (pl/s)					
Mean ± SD	175 ± 64	175 ± 79	195 ± 61	173 ± 94	0.5
[Min–Max]	[3–3197]	[3–7855]	[3–5408]	[3–7937]	
WSR (s^−1^)					
Mean ± SD	183 ± 56	154 ± 47	164 ± 52	153 ± 52	**0**.**05**
[Min–Max]	[15–2218]	[17–1406]	[10–2722]	[13–1405]	
WSS (dyne/cm^2^)					
Mean ± SD	4.8 ± 1.8	3.8 ± 1.2	3.9 ± 1.6	3.6 ± 1.3	**0**.**005**
[Min–Max]	[0.03–9.88]	[0.03–4]	[0.02–9.5]	[0.02–5.6]	
Venules sample size					
Mean ± SD	52 ± 21	59 ± 18	59 ± 23	52 ± 16	0.1
[Min–Max]	[14–108]	[26–101]	[19–150]	[18–79]	

C, NDR, NPDR, and PDR are abbreviations for non-diabetic control, no clinically visible diabetic retinopathy, non-proliferative diabetic retinopathy, and proliferative diabetic retinopathy, respectively. D, V, Q, WSR, and WSS are abbreviations for diameter, axial blood velocity, blood flow, wall shear rate and wall shear stress, respectively. Bold indicates statistical significance. The ± symbol refers to standard deviation (SD).

*P-value determined by ANOVA.

**Table 6:** Comparison of conjunctival hemodynamic descriptors in venules between C and DR subjects in unadjusted (Model 1) and adjusted (Model 2) models.

	D (μm)		V (mm/s)		Q (pl/s)		WSR (s^−1^)		WSS (dyne /cm^2^)	
	β	P	β	P	β	P	β	P	β	P
Intercept	20	<0.001	0.59	<0.001	173	<0.001	183	<0.001	4.8	<0.001
**Model 1**: DR Stage Effect*										
C	Ref.	Ref.	Ref.	Ref.	Ref.	Ref.	Ref.	Ref.	Ref.	Ref.
NDR	1.2	**0**.**03**	−0.05	0.2	5	0.8	−29	**0**.**01**	−1.1	**0**.**001**
NPDR	1.4	**0**.**02**	−0.01	0.7	25	0.1	−19	0.1	−0.9	**0**.**008**
PDR	0.4	0.52	−0.06	0.1	4	0.8	−29	**0**.**02**	−1.2	**<0**.**001**
Intercept	19	<0.001	0.49	0.004	151	0.05	185	**0.001**	3.4	**0.03**
**Model 2**: DR Stage Effect**										
C	Ref.	Ref.	Ref.	Ref.	Ref.	Ref.	Ref.	Ref.	Ref.	Ref.
NDR	1.4	**0**.**03**	−0.06	0.2	7	0.7	−33	**0**.**01**	−1.1	**0**.**004**
NPDR	2.1	**0**.**006**	−0.03	0.6	26	0.2	−29	**0**.**05**	−1.1	**0**.**02**
PDR	1.1	0.19	−0.11	**0**.**04**	−6	0.8	−47	**0**.**003**	−1.4	**0**.**002**

C, NDR, NPDR, and PDR are abbreviations for non-diabetic control, no clinically visible diabetic retinopathy, non-proliferative diabetic retinopathy, and proliferative diabetic retinopathy, respectively. D, V, Q, WSR, and WSS are abbreviations for diameter, axial blood velocity, blood flow, wall shear rate and wall shear stress, respectively. Bold indicates statistical significance.

*Unadjusted.

**Adjusted for age, race, sex, mean arterial pressure, heart rate, hematocrit, and hemoglobin A1C.

**Table 7:** Comparison of association between conjunctival V and D in venules between C and DR subjects in unadjusted (Model 1) and adjusted (Model 2) models.

	Slope (s^−1^)	95% CI	P-value*
**Model 1**: **DR Stage Effect**			
C	**18**^a^	**16**–**21**	Ref
NDR	**18**^a^	**16**–**19**	0.6
NPDR	**22**^a^	**20**–**23**	**0**.**01**^b^
PDR	**21**^a^	**19**–**23**	0.1
**Model 2**: **DR Stage Effect**			P-value**
C	**18**^a^	**16**–**21**	Ref
NDR	**18**^a^	**16**–**19**	0.5
NPDR	**22**^a^	**20**–**23**	**0.01**^**b**^
PDR	**21**^a^	**19**–**23**	0.1

Slopes of the regression lines relating V and D are provided. C, NDR, NPDR, and PDR are abbreviations for non-diabetic control, no clinically visible diabetic retinopathy, non-proliferative diabetic retinopathy, and proliferative diabetic retinopathy, respectively. Bold indicates statistical significance.

^a^Significant association between V and D.

^b^Association between V and D significantly different than C (ref).

*Unadjusted.

**Adjusted for age, race, sex, mean arterial pressure, heart rate, hematocrit, and hemoglobin A1C.
